# A Discovery of Relevant Hepatoprotective Effects and Underlying Mechanisms of Dietary *Clostridium butyricum* Against Corticosterone-Induced Liver Injury in Pekin Ducks

**DOI:** 10.3390/microorganisms7090358

**Published:** 2019-09-16

**Authors:** Yanhan Liu, Cun Liu, Liqing Huang, Zhaofei Xia

**Affiliations:** 1 College of Veterinary Medicine, China Agricultural University, Beijing 100193, China; vetlyh@163.com (Y.L.); hlqcau@126.com (L.H.); 2 Institute of Animal Sciences, Chinese Academy of Agricultural Sciences, Beijing 100193, China; liucun89@163.com

**Keywords:** corticosterone, *Clostridium butyricum*, liver, RNA-seq, Pekin ducks

## Abstract

*Clostridium butyricum* (*C. butyricum*) can attenuate oxidative stress, inflammation, and hepatic fatty deposition in poultry, however, the underlying mechanisms for this in Pekin ducks remain unclear. This study evaluated these hepatoprotective effects and the underlying mechanisms in a corticosterone (CORT)-induced liver injury model in Pekin ducks fed a *C. butyricum* intervention diet. A total of 500 Pekin ducks were randomly divided into five groups: one group (CON group) was only provided with a basal diet, three groups were provided a basal diet with 200 mg/kg (LCB group), 400 mg/kg (MCB group), or 600 mg/kg (HCB group) *C. butyricum*, respectively, and one group was provided a basal diet with 150 mg/kg aureomycin (ANT group) for 42 d. At 37 days-old, all ducks received daily intraperitoneal injections of CORT for five days to establish a liver injury model. *C. butyricum* intervention alleviated liver injury by decreasing the liver organ indices, hepatic steatosis and hepatocyte necrosis, and improving liver function, antioxidant capacity, and inflammatory factors. Hepatic RNA-seq revealed 365 differentially expressed genes (DEGs) between the MCB and CON groups, with 229 up- and 136 down-regulated DEGs in the MCB group. Between the MCB and ANT groups, 407 DEGs were identified, including 299 up- and 108 down-regulated genes in MCB group. Some DEGs in the MCB group related to oxidative stress and inflammatory responses such as Sod3, *Tlr2a/b*, and *Il10,* which were up-regulated, while *Apoa1*, *Cyp7a1*, *Acsl1/5*, *Fasn*, *Ppar-γ*, and *Scd*, which are involved in lipid metabolism, were down-regulated, indicating that these genes were responsive to dietary *C. butyricum* for the alleviation of corticosterone-induced hepatic injury. Toll-like receptor signaling, PI3K-Akt signaling pathway, cytokine-cytokine receptor interaction, peroxisome proliferator-activated receptor (PPAR) signaling pathway, adipocytokine and glycerophospholipid metabolism signaling pathway were significantly enriched in the MCB group. These findings indicate that *C. butyricum* intervention can protect Pekin ducks from corticosterone-induced liver injury by the modulation of immunoregulatory- and lipid metabolism-related genes and pathways.

## 1. Introduction

The timid nature of Pekin ducks makes them easily stressed. Intensively reared ducks are constantly exposed to various adverse influences, such as noise, crowding, handling, transport, high temperature, and immune challenges, which leads to chronic stress [1,2]. Stress in poultry can activate the hypothalamic pituitary adrenal axis, resulting in increased corticosterone (CORT) secretion [3]. Therefore, the serum CORT level is considered a reliable stress indicator in poultry [4]. Recently, numerous publications have demonstrated that a CORT challenge can reliably induce physiological stress in rodents and birds [5,6]. Additionally, a CORT challenge can also lead to decreased body weight, and can influence lipid metabolism and immune system responses [7,8,9].

The liver plays an important role in maintaining metabolic homeostasis [10]. A fatty liver, oxidative stress, and inflammation are the major mechanisms underlying the pathogenesis of liver injury induced by CORT [8,11,12]. Hepatic inflammation and lipogenesis can be affected by a number of hormones, including glucocorticoids, which modulate metabolic homeostasis and stress responses [13,14]. Glucocorticoids are important regulators of lipid metabolism, and chronic low level exposure to glucocorticoids promotes lipogenesis to produce fatty liver [15,16]. CORT is the primary active form of glucocorticoids and is considered as an induced indicator of fatty liver and stress [17]. Chronic high levels of CORT are closely related to fatty liver progression and inflammatory responses [18]. Exogenous CORT administration to animals can induce abnormal lipid accumulation and an inflammatory response in the liver that can progress to fatty liver diseases [19,20]. However, no relevant studies in Pekin ducks have been reported in the literature.

*Clostridium butyricum* (*C. butyricum*), regarded as a beneficial probiotic, exists in the intestinal tract and acts to protect against pathogenic bacteria and intestinal injury [21]. Direct feeding of *C. butyricum* to different animals (such as broilers, Pekin ducks and shrimps) can enhance growth performance, antioxidant properties, as well as meat quality, and modulate intestinal microflora, balance intestinal health, and regulate the immune system by activating related genes and signaling pathways [22,23,24]. Beyond its beneficial effects in the digestive tract, *C. butyricum* can protect mice against non-alcoholic fatty liver disease (NAFLD) induced by a high fat diet [25] and the anti-oxidation and anti-inflammation protective effects of *C. butyricum* have also been demonstrated in previous studies for other diseases [26,27]. Although the probiotic *C. butyricum* can improve the intestinal health and immune status of weaned piglets challenged with lipopolysaccharide (LPS) [28] and suppress hepatic damage degree in mice challenged with CORT [25], it is largely unknown whether probiotic *C. butyricum* intervention can attenuate liver injury in Pekin ducks under CORT challenge. Transcriptome analysis is an effective technique to identify the genes that are differentially expressed and signaling pathways that are affected by environmental endocrine disruptors and we have already used transcriptome analysis to demonstrate the effects of dietary *C. butyricum* on the breast muscle of Pekin ducks [29]. Therefore, it is interesting to use the RNA-sequencing (RNA-seq) method in order to determine the underlying hepatoprotective mechanisms active in Pekin ducks supplemented with dietary *C. butyricum* under CORT challenge through a combined analysis of lipid metabolism and inflammation response.

In this study, we aimed to investigate whether dietary *C. butyricum* intervention can alleviate CORT-induced oxidative stress, inflammation, and lipid accumulation in the liver of Pekin ducks, and to explore the underlying mechanisms of these effects using the RNA-sequencing technique. We hope our study can help to solve the stress problems of Pekin duck in the breeding process for the future, and eventually enhance the associated economic income.

## 2. Materials and Methods

### 2.1. Ethics Statement

This study was carried out in accordance with the recommendations of Animal Care and Use Ethics Committee of China Agricultural University under protocol code CAU20180428-2. The protocol was approved by the Animal Care and Use Ethics Committee of China Agricultural University (approval date: 28 April 2018).

### 2.2. Animals and Experimental Design

**Figure 1:**
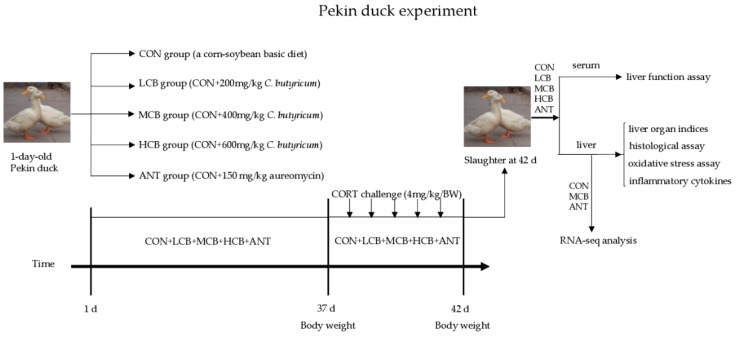
The Pekin duck experiment design and the subsequent assays.

A total of 500 male Pekin ducks (1-day-old) were selected from a local commercial hatchery (Beijing Golden Star Duck Co., Ltd.) and the experiment was performed at the Experimental Center of China Agricultural University. The birds were randomly assigned into five groups (five replicates with twenty birds each) and were raised in a temperature-controlled room. The temperature was controlled at 32 to 34 °C during the first 5 days, and then subsequently declined (about 2–3 °C/week) to a final temperature of 22 °C. All ducks had free access to feed and drinking water with a 23L: 1D lighting system during the experiment. The control group (CON) was fed with a corn-soybean basic diet for 42 d. The other four groups were fed with the basic diet supplemented with 200 mg/kg (LCB), 400 mg/kg (MCB), or 600 mg/kg (HCB) *C. butyricum* (2.0 × 10^9^ CFU/g) or 150 mg/kg aureomycin (ANT), respectively (Figure 1). The nutrient requirements of the diets were calculated to meet or exceed the National Research Council (NRC, 1994) standards (Table S1). The *C. butyricum* is available as a freeze-dried powder obtained from Beijing Shine Biology Technology Co., Ltd., China (Batch No. 20170325003).

At 37-days-old, twelve ducks were randomly selected from each group and challenged with CORT (4 mg/kg, Sigma-Aldrich, Shanghai, China) for 5 days. CORT was premixed in corn oil (1:1) and injected into the abdominal cavity. The dose of 4 mg/kg was demonstrated previously to enhance serum CORT content. Injections were done once daily between 07:00 and 08:00.

### 2.3. Sample Collection

The body weight of the selected 12 ducks from each group was recorded after feed fasting for 6 h at 37- and 42-days old. On day 42, blood samples (5 mL) were taken from the wing vein for biochemical analysis. Subsequently, all 12 ducks were euthanized by giving sodium pentobarbitone solution (30 mg/kg/BW) intravenously through the leg anesthetic to reduce discomfort. After dissection, the entire liver was weighed to calculate the liver organ indices. All liver samples were frozen in liquid nitrogen immediately and stored at –80 °C until further RNA-seq analysis.

### 2.4. Histological Examination of Hepatic Tissue

Liver samples were freshly dissected and, after appropriate fixation (10% formaldehyde neutral buffer solution) and paraffin embedding, samples were sectioned at a thickness of 4 μm and stained with hematoxylin and eosin (H&E). Frozen liver sections were made at −18 °C using a Leica kryostat (Leica CM3050S, Leica instrument GmbH, Germany), then stained with Oil Red O (O0625; Sigma-Aldrich Co., St. Louis, MO, USA) for 15 min and counterstained with hematoxylin. The slides were observed using an optical microscope (Olympus BX50 microscope; Olympus Corporation, Tokyo, Japan).

### 2.5. Measurement of Liver Function Indices

The levels of aspartate aminotransferase (AST), alanine aminotransferase (ALT), and gamma-glutamyltranspeptidase (γ-GGT) in serum were measured using the assay kits (Nanjing Jiancheng Inc., Nanjing, China) according to the manufacturer’s instructions. The total protein (TP), albumin (ALB), and globulin (GLB) were detected using a Roche Cobas702 automatic biochemical analyzer (Roche, Basel Switzerland).

### 2.6. Detection of Oxidative Stress and Cytokines

Liver tissues (200 mg) were homogenized in 0.9% physiological saline to obtain a 10% homogenate. The total antioxidant capacity (T-AOC), total superoxide dismutase (T-SOD), and glutathione peroxidase (GSH-PX) activities as well as the reactive oxygen species (ROS) and malondialdehyde (MDA) contents of the 10% homogenate were measured using commercial kits (Nanjing Jiancheng Bioengineering Institute, Nanjing, China). The total hepatic protein was detected by the method of Coomassie brilliant blue using the commercial kit (Nanjing Jiancheng Bioengineering Institute, China). The concentrations of interleukin-1β (IL-1β), tumor necrosis factor-α (TNF-α), IL-6, IL-4, IL-10 and transforming growth factor-β (TGF-β) in the liver homogenate were detected using commercial ELISA kits (Nanjing Jiancheng Bioengineering Institute, China). All procedures were performed according to the manufacturer’s instructions. The levels of CORT in serum were determined by the Beijing Huaying Biotechnology Company (Beijing, China) using a radioimmunoassay method.

### 2.7. RNA Isolation, Library Preparation, and Sequencing

The liver samples of nine randomly selected ducks (three from each of the CON, MCB, and ANT groups) were separately ground for 30 s using the easy grind method in liquid nitrogen. Total RNA was then extracted using TRIzol reagent (Invitrogen, Carlsbad, CA, USA) following the manufacturer’s protocol. RNA quality and purity were determined using a Nano-Drop 2000 spectrophotometer (NanoDrop Technologies, Wilmington, DE, USA) at 260 and 280 nm, and RNA integrity was checked on an Agilent 2100 Bionalyzer (Agilent Technologies, Les Ullis, France) and by 1.2% agarose gel electrophoresis. Only RNA samples with OD_260_/OD_280_ ratios between 1.8 and 2.2 and an RNA integrity number (RIN) ≥ 8.0 were selected for cDNA library generation. Next, RNA samples from the nine ducks were then packed in dry ice and sent to Shanghai Majorbio Bio-pharm Technology Co., Ltd. (Shanghai, China) for further library preparation and sequencing. The cDNA library was sequenced using an Illumina HiSeq 4000 (Illumina, San Diego, CA, USA) using paired-end technology.

The quality of raw reads was evaluated using the FastQC program (http://www.bioinformatics.babraham.ac.uk/projects/fastqc/). Raw reads with a threshold quality < 20, reads shorter than 50 nt, as well as reads containing adapter sequences, poly-N, or the sequencing primer were removed using SeqPrep software (https://github.com/jstjohn/SeqPrep) and Sickle software (https://github.com/najoshi/sickle) to obtain clean reads. Additionally, the quality of the clean data was evaluated according to Error%, Q30, GC content%, average read length and sequence duplication level. The high-quality clean reads were used for subsequent analyses and functional annotation. The high quality clean reads of the nine samples were separately mapped to the reference *Anas platyrhynchos* genome (http://asia.ensembl.org/Anas_platyrhynchos/Info/Index?db=core) using TopHat2 (version: 2.0.9) [30]. The normalization of gene expression levels was obtained by converting the RNA-seq transcript abundance into fragments per kilobase per million mapped reads (FPKM) [31]. The RNA-seq datasets used and/or analyzed during the study are available in the NCBI Sequence Read Archive (SRA) under the accession number SRP134223.

### 2.8. Enrichment Analysis of GO Terms and KEGG Pathways for DEGs

The edgeR software (http://www.bioconductor.org/packages/2.12/bioc/html/edgeR.html) was used to identify differentially expressed genes (DEGs) among the three groups and fold change was calculated based on FPKM values. The *p* values were adjusted by a false discovery rate (FDR) correction method. Genes with a corrected *p*-value < 0.05 and fold-change ≥ 2 or ≤ 0.5 were considered to be significantly differentially expressed.

Gene ontology (GO) term enrichment analysis and Kyoto Encyclopedia of Genes and Genomes (KEGG) pathway enrichment analysis for the identified DEGs were performed using the free online platform Majorbio I-Sanger Cloud Platform (www.i-sanger.com) to predict the metabolic pathways in which the DEGs are involved. The DEGs of GO term enrichment was classified into the categories of molecular functions, cellular component, and biological process. A corrected *p*-value < 0.05 indicated significant enrichment. Additionally, a complementary and more comprehensive pathway activation strength (PAS) value analysis of GO processes based on DEGs was performed using OmicsBean (http://www.omicsbean.cn) to further identify the deep biological processes and pathways.

### 2.9. Confirmation of RNA-seq Data by qRT-PCR

To validate the accuracy of the RNA-seq data, we randomly selected 20 DEGs for qRT-PCR analysis. A total of six RNA samples from each group were reverse transcribed to complementary DNA using a Prime Script RT Master Mix Kit with DNase I (TaKaRa, Dalian, China) following the manufacturer’s instructions. Primers were designed using primer premier 5.0 software and were synthesized by Sangon Biotech Co., Ltd. (Shanghai, China) (Table S2). Then, qRT-PCR was performed in triplicate reactions in 96 well plates using the Top Green qPCR SuperMix Kit (TransGen Biotech, Beijing, China) according to the manufacturer’s instructions using the following cycling conditions: 95 °C for 30 s, followed by 40 cycles of 95 °C for 10 s, and 55 °C for 42 s, then 72 °C for 10 s, followed by 72 °C for 5 min. A melting curve was then produced using an ABI QuantStudio 7 Flex Sequence Detection System (Applied Biosystems Co. Ltd., Foster City, CA, USA). The fold expression change of DEGs was calculated using the 2^−ΔΔCt^ method [32]. The mRNA levels of the DEGs were normalized against an endogenous reference gene, glyceraldehyde-3-phosphate dehydrogenase (*Gapdh*).

### 2.10. Statistical Analysis

The results are presented as the mean ± standard deviation (S.D.). The data were determined by one-way ANOVA followed by Duncan’s multiple comparisons between groups using SPSS25.0 software (SPSS, Chicago, IL, USA). *p* < 0.05 or less was considered statistically significant.

## 3. Results

### 3.1. C. butyricum Alleviated Impaired Growth Performance and Liver Organ Indices Induced by CORT

**Figure 2:**
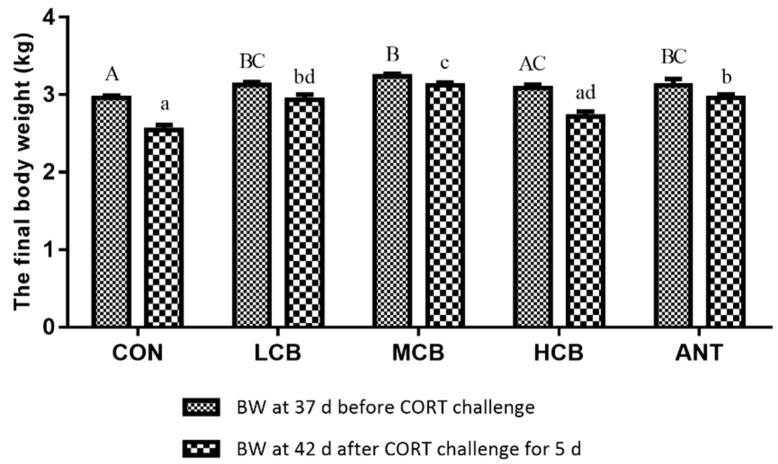
Effects of *C. butyricum* on body weight (BW) of Pekin ducks at 37 d before corticosterone (CORT) challenge and 42 d after CORT challenge for 5 d, respectively. The data were showed as mean ± S.D. A, B, C was used to show the differences among groups at 37 d before CORT challenge and the different superscript capital letter indicated significant difference (*p* < 0.05) between any two groups at 37 d before CORT challenge. a, b, c, d was used to show the differences among groups at 42 d after CORT challenge for 5 d and the different superscript small letter indicated significant difference (*p* < 0.05) between any two groups at 42 d after CORT challenge for 5 d.

The effects of *C. butyricum* intervention on body weight (BW) of Pekin ducks were presented in Figure 2. On day 37, before the CORT challenge, the final body weights in LCB, MCB, and ANT groups were significantly (*p* < 0.05) greater than that in the CON group. On day 42, 5 days after the CORT challenge, the final body weights in LCB, MCB, and ANT groups were still significantly (*p* < 0.05) greater than that in the CON group. Supplementing ducks with *C. butyricum* at 600 mg/kg had no impact on body weight either before or after the CORT challenge. The CORT challenge for five days decreased the final body weight of all Pekin ducks, however, *C. butyricum* intervention could alleviate the decrease of body weight. Additionally, CORT challenge for five days significantly increased the liver organ indices in the CON group, but this increase significantly decreased in the MCB and HCB groups at 42 d (Table 1).

### 3.2. C. butyricum Alleviated Liver Function Indices Induced by CORT

As shown in Table 2, CORT treatment increased the serum activities of AST and ALT at 42 d (*p* < 0.05). However, *C. butyricum* intervention significantly alleviated the CORT-increased levels of AST and ALT (*p* < 0.05). CORT treatment decreased ALB contents (*p* < 0.05) while *C. butyricum* intervention significantly (*p* < 0.05) prevented this decrease at 42 d. Thus, the probiotic *C. butyricum* can protect against CORT-induced liver damage.

### 3.3. C. butyricum Alleviated Pathological Liver Damage Induced by CORT

**Figure 3:**
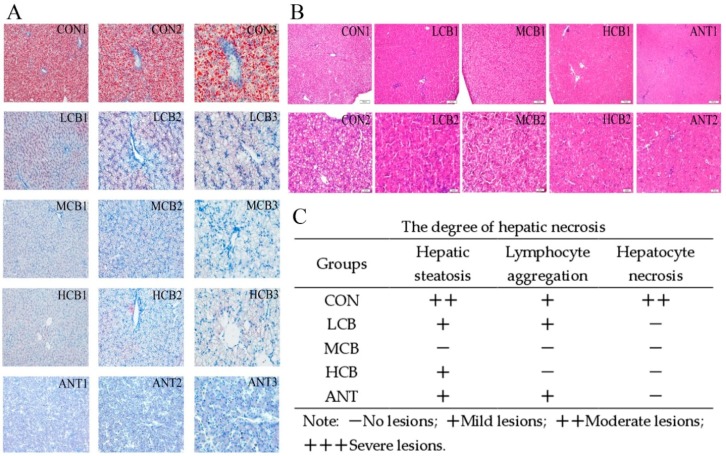
The effects of *C. butyricum* on histological changes and lipid distribution in the livers of Pekin ducks after CORT challenge. (**A**) Oil red O staining (1: 100×, 2: 200×, 3: 400×). (**B**) Hematoxylin-eosin (H&E) staining. (**C**) The effects of *C. butyricum* on the protective degree of hepatic stestosis, lymphocyte and hepatocyte necrosis in different groups after CORT challenge.

The histological sections stained with hematoxylin-eosin (H&E) (Figure 3A) and Oil Red O (Figure 3B) both indicated that lipid accumulation severely increased at 42 d after the CORT challenge, but this increase was drastically attenuated in the *C. butyricum*-supplemented groups and the ANT group. Additionally, hepatic steatosis, lymphocyte aggregation, severe hepatocyte necrosis, and inflammatory cell infiltration especially around the central veins were observed in the CON group liver after CORT challenge. However, these pathological changes were clearly attenuated by *C. butyricum* intervention, especially in the MCB group. The relieved degree of hepatic steatosis, lymphocyte aggregation and hepatocyte necrosis were presented in the Figure 3C.

### 3.4. C. butyricum Alleviated Hepatic Oxidative Stress Induced by CORT

The CORT challenge weakened the activities of T-SOD and GSH-PX while it increased ROS and MDA levels in the liver and CORT concentration in serum. However, *C. butyricum* intervention had the opposite effects, as it significantly enhanced the activities of T-SOD and GSH-PX and reduced the ROS and MDA levels (*p* < 0.05), especially in MCB and HCB at 42 d. Moreover, the CORT contents in the *C. butyricum*-intervened groups significantly (*p* < 0.05) decreased compared to the CON group at 42 d (Table 3).

### 3.5. C. butyricum Alleviated the Hepatic Inflammation Response Induced by CORT

To investigate whether inflammation could be attenuated by *C. butyricum*, the levels of pro-inflammatory cytokines (IL-1β, IL-6 and TNF-α) in liver tissue at 42 d were tested (Table 4). The CORT challenge clearly increased the concentrations of IL-1β, IL-6 and TNF-α in the liver, but these increases were significantly decreased by *C. butyricum* intervention (*p* < 0.05). Moreover, we also detected the production of the anti-inflammatory factors IL-4, IL-10, and TGF-β in the liver, and found that they were all reduced by the CORT challenge, but that IL-4 and IL-10 were significantly elevated (*p* < 0.05) by *C. butyricum*.

### 3.6. Quality Control of Liver RNA-seq Data

We established nine cDNA libraries from the livers of ducks at 42 d from the CON, MCB, and ANT groups, with three replicates in each group. Quality control is an essential step to determine the quality of RNA-seq data. RNA-seq generated 56781984-86256452 raw reads for each library (Table 5). Low-quality reads were filtered out, and the average numbers of clean reads were 69861501 (95.70%), 69132671 (97.53%), and 67804130 (97.59%) of the CON, MCB and ANT groups, respectively. We only used the clean reads for downstream analyses. After assembly, an average of 72.77% clean reads from each library was uniquely mapped to the reference duck genome (http://asia.ensembl.org/Anas_platyrhynchos/Info/Index?db=core), and the average mapping ratios were 69.85%, 76.53%, and 71.94% for the CON, MCB and ANT groups, respectively. The error rate, GC (guanine-cytosine) content, Q20 (Phred quality score 20), Q30, and mapping ratio of each samples are listed in Table 6. We concluded that the RNA-seq data was of reliable quality. The mapped reads in different regions of the genome are presented in Figure S1. The 15 most abundantly expressed genes in both MCB and ANT groups, ranked by absolute abundance, were *Alb*, *Ttr*, *Thrsp*, *Hpx*, *Apoa1*, *Ahsg*, *Mt1*, *Eee1a1*, *Vtn*, *Fabp1*, *Ppdpf*, *Fgg*, *Aldob*, *Fgb*, and *Scd* (Table S3). The GO categories and KEGG analysis of functional annotation and classification related to all unigenes are shown in Supplementary Figures S2 and S3. Through the alignment of GO and KEGG databases, the largest number of genes was associated with signal transduction and cellular process.

### 3.7. Identification of DEGs

**Figure 4:**
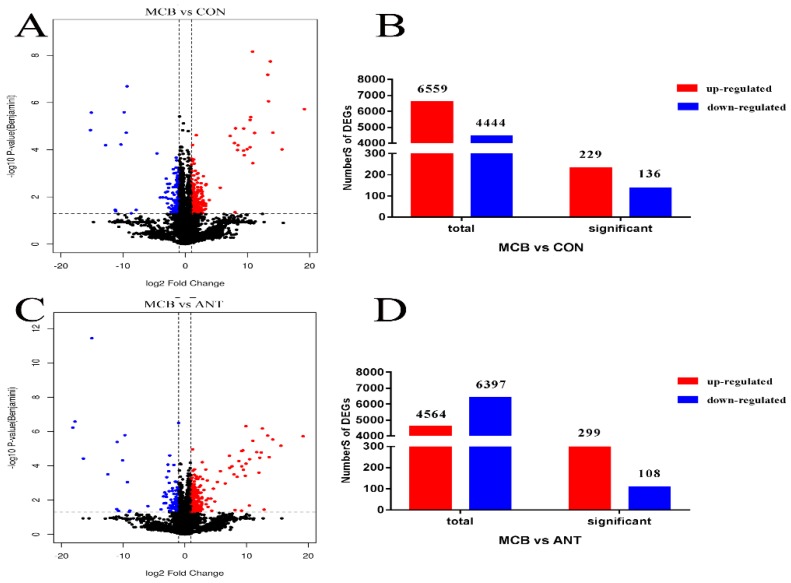
The volcano plot and numbers of DEGs. (**A**) The volcano plot between the MCB and CON groups. (**B**) The numbers of the DEGs between the MCB and CON groups. (**C**) The volcano plot between the MCB and ANT groups. (**D**) The numbers of the DEGs between the MCB and ANT groups. (**A**,**C**): Red dots (Up) represent significantly up-regulated genes (*p* < 0.05, fold change ≥ 2); blue dots (Down) represent significantly down-regulated genes (*p* < 0.05, fold change ≤ 0.5); black dots (No) represent insignificantly DEGs. (**B**,**D**): The first two columns represent the total number of up-regulated genes (*p* < 0.05 and *p* ≥ 0.05, fold change ≥ 2) or down-regulated genes (*p* < 0.05 and *p* ≥ 0.05, fold change ≤ 0.5), respectively; the second two columns represent the number of up-regulated genes (*p* < 0.05, fold change ≥ 2) or down-regulated genes (*p* < 0.05, fold change ≤ 0.5), respectively.

**Figure 5:**
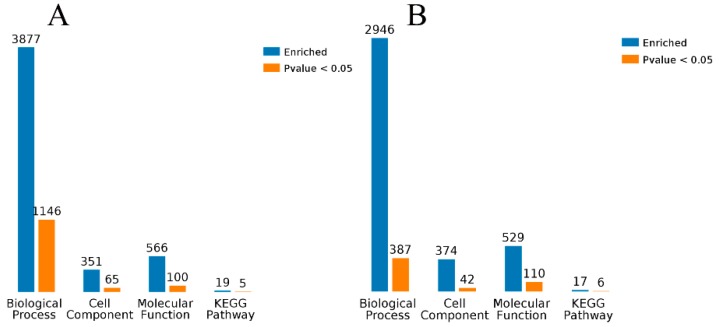
Biological process, cellular component, molecular function and KEGG pathway are the four enrichment categories that were used for the functional analysis of DEGs from the MCB vs. CON groups (**A**) and the MCB vs. ANT groups (**B**), respectively. Blue bars: Counts for each category represent the total associated terms in the database with the query gene/protein list. Orange bars: Terms with *p*-value < 0.05 is statistically significant.

The volcano plot of the significantly DEGs was shown in Figure 4A,C. A total of 365 significantly DEGs (229 up-regulated and 136 down-regulated) were identified between the MCB and CON groups (Figure 4B; Supplementary file 1; FDR ≤ 0.05, fold-change ≥ 2 or ≤ 0.5) using edgeR software. The numbers of significantly enriched genes in the terms biological process, cell component, molecular function, and the KEGG pathway were 1146, 65, 100, and 5, respectively (Figure 5A). A total of 407 significantly DEGs (299 up-regulated and 108 down-regulated) were identified between the MCB and ANT groups (Figure 4D; Supplementary file 1; FDR ≤ 0.05, fold-change ≥ 2 or ≤ 0.5) using edgeR software. The numbers of significantly enriched genes in the terms biological process, cell component, molecular function, and the KEGG pathway were 387, 42, 110, and 6, respectively (Figure 5B).

### 3.8. GO and KEGG Analysis of DEGs in Liver

**Figure 6:**
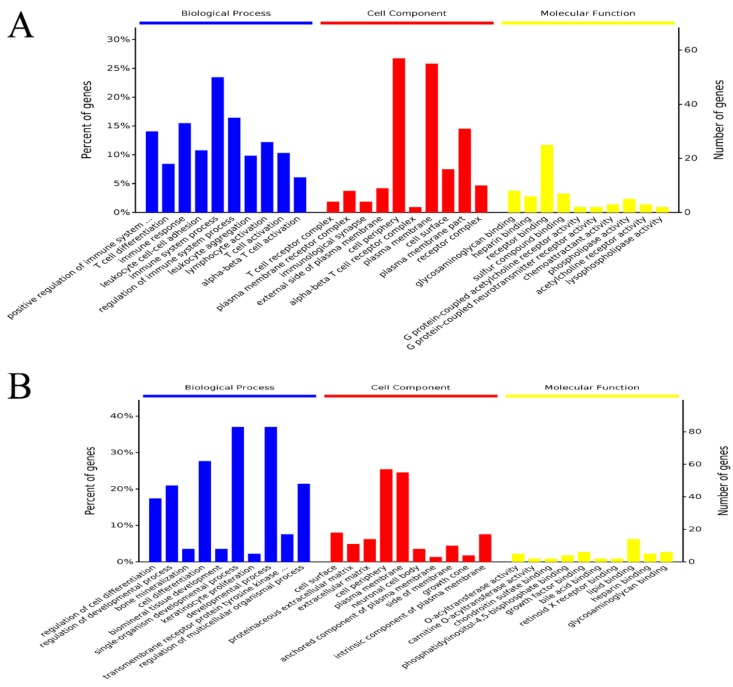
The top ten enriched terms of DEGs in each main category (Biologic Process, Cell Component, and Molecular Function) of the GO database. The right y-axis represents the number of genes involved in each GO annotation, and the left y-axis represents the percent of annotated genes in each term to the total number of DEGs. (**A**) Enriched terms based on DEGs between the MCB and CON groups. (**B**) Enriched terms based on DEGs between the MCB and ANT groups.

**Figure 7:**
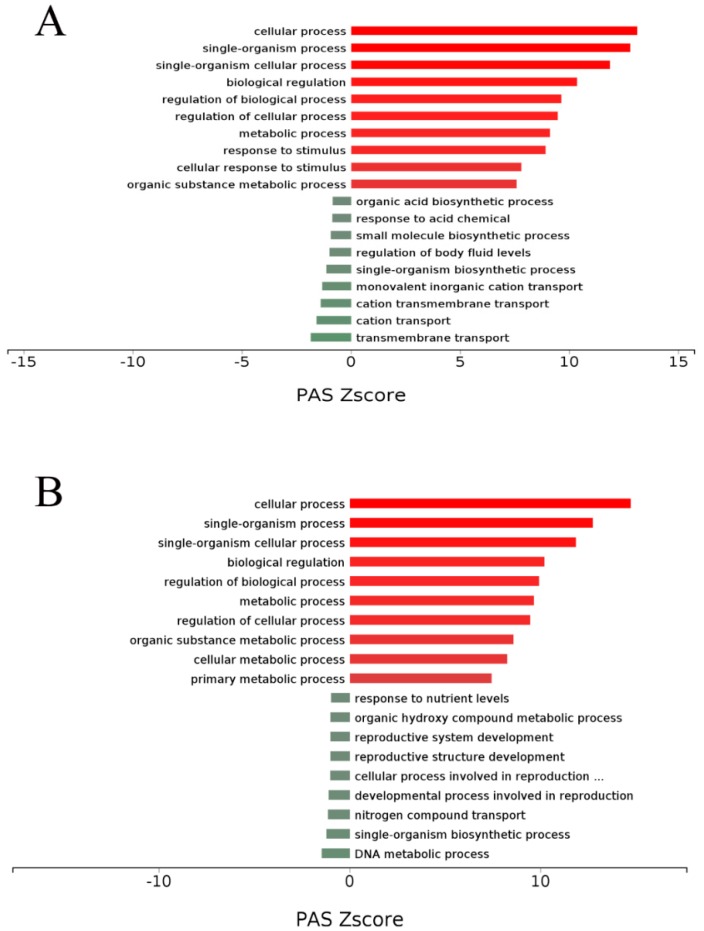
PAS value of primary enriched GO processes of DEGs using the OmicsBean system. (**A**) Based on the DEGs between the MCB and CON groups. (**B**) Based on the DEGs between the MCB and CON groups. Differentially enriched GO processes were identified according to their reporter score from the Z-score of individual genes. A reporter score of Z = 1.96 or Z = −1.96 was used as a detection threshold for significantly different processes.

We used DEGs to further analyze GO and KEGG enrichment in order to determine their potential functions and metabolic pathways. The DEGs between the CON and MCB groups were primarily enriched in the positive regulation of immune system process, T cell differentiation, immune response, leukocyte cell-cell adhesion, immune system process, regulation of immune system process, leukocyte aggregation, lymphocyte activation, T cell activation, alpha-beta T cell activation, and lipid metabolic process in molecular functions (Figure 6A; Supplementary file 2). Therefore, the GO term enrichment analysis showed that the DEGs between the CON and MCB groups were significantly enriched in immunity and lipid metabolism. The DEGs between the MCB and ANT groups were primarily enriched in the regulation of cell differentiation, regulation of developmental process, cell differentiation, single-organism developmental process, developmental process, regulation of multicellular organismal process, cellular lipid metabolic process, and lipid metabolic process in molecular functions (Figure 6B; Supplementary file 2). The GO term enrichment analysis showed that the DEGs between the MCB and ANT groups were significantly enriched in development and metabolism. The primary enriched GO processes of DEGs are shown in Figure 7. Compared with the CON group, providing a moderate amount of *C. butyricum* to Pekin ducks mainly up-regulated the cellular process, metabolism process, and response to stimulus and down-regulated the biosynthetic process in the liver (Figure 7A). However, the cellular process and metabolism process were up-regulated, and the developmental process involved in reproduction was down-regulated in the MCB group compared with the ANT group (Figure 7B). Therefore, moderate levels of *C. butyricum* had a protective effect on the CORT-induced liver damage compared with the CON and ANT groups.

In addition, compared with the CON group, *C. butyricum* dietary supplementation at 400 mg/kg significantly (*p* < 0.05) activated cell adhesion molecules (CAMs), cytokine-cytokine receptor interaction, PI3K-Akt signaling pathway, metabolism of xenobiotics by cytochrome P450, PPAR signaling pathway, glutathione metabolism, and Toll-like receptor signaling pathway in the liver. Dietary supplementation with *C. butyricum* activated glycerophospholipid metabolism, AMP-activated protein kinase (AMPK) signaling pathway, cytokine-cytokine receptor interaction, PPAR signaling pathway, adipocytokine signaling pathway, and fatty acid biosynthesis in the liver (Table 6; Supplementary file 3). The DEGs enriched in the above pathways are presented in Table 7. Our KEGG pathway enrichment analysis of the DEGs showed that many immunity and lipid metabolism-related pathways were significantly enriched for DEGs in the alleviation of CORT-induced liver damage in Pekin ducks supplemented with *C. butyricum*.

### 3.9. Confirmation of Gene Expression with Quantitative RT-PCR

**Figure 8:**
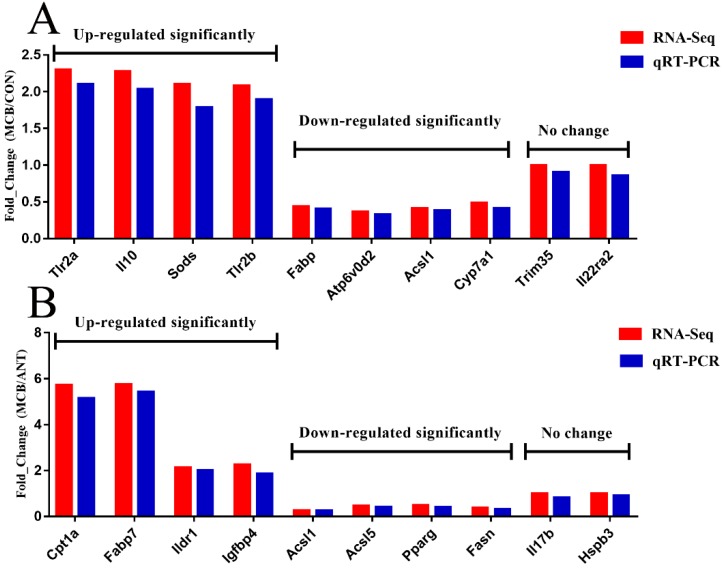
Validation of DEGs by qRT-PCR. *Gapdh* was used as an internal control, and data are presented as fold change (*N* = 6 per group). (**A**) The DEGs between the MCB and CON groups. (**B**) The DEGs between the MCB and ANT groups.

To confirm the accuracy of the RNA-seq transcriptome data, we randomly selected ten genes between the MCB and CON groups and ten genes between the MCB and ANT groups—each ten genes including four significantly up-regulated genes, four significantly down-regulated genes, and two genes with no significant differential expression. The expression levels of the selected genes were quantified using qRT-PCR, and the results were consistent with the transcriptome results (Figure 8). The results suggest that RNA-Seq reliably identified the DEGs in the duck’s liver transcriptome.

## 4. Discussion

*C. butyricum* is reported to have a variety of beneficial effects in humans and animals, including promoted growth performance [33], improved meat quality [22], enhanced immune function [34], alleviated oxidative stress [26], attenuated fatty liver disease [25], and balanced intestinal microflora [33]. In our study, *C. butyricum* supplementation at 200mg/kg (LCB) and 400 mg/kg (MCB) both significantly increased the body weight of Pekin ducks at 37 d before CORT challenge. However, *C. butyricum* supplementation at 600 mg/kg (HCB) did not significantly affect growth performance compared with the CON group, indicating that 600 mg/kg *C. butyricum* does not positively promote the growth performance of Pekin ducks. Antibiotic supplementation also had positive effects on Pekin duck’s growth performance. Numerous studies have reported that sub-therapeutic use of antibiotics in diets can promote growth performance and control gastrointestinal bacterial infections in Pekin ducks [35]. However, the use of antibiotics which has largely led to the emergence of resistant bacteria and the potential for producing drug residues in animal feed and products; therefore, a ban has been issued [36]. *C. butyricum* is, therefore, an alternative to improve the growth performance of animals. Exogenous CORT administration significantly suppresses body weight in ducks [2], which is consistent with the results of our study. In our study, the body weight of ducks at 42 d decreased after CORT challenge, but *C. butyricum* intervention (especially 400 mg/kg) significantly attenuated this degree. Although the antibiotic presented the same protective effects as *C. butyricum*, we recommend using *C. butyricum* instead of antibiotics.

The liver is susceptible to CORT challenges, so it was selected as the research model. To further confirm the protective effects of *C. butyricum* on hepatic function in Pekin ducks, we measured the serum levels of aminotransferase protein and pathological changes in the liver, which are classical parameters used to evaluate hepatic damage induced by CORT [20]. The serum ALT level is used as a special parameter of hepatotoxic effects, while AST activity is considered a less specific biomarker of liver function. However, the elevated AST and ALT levels induced by CORT were drastically attenuated by *C. butyricum* intervention, and the 400 mg/kg *C. butyricum* intervention had the best effects. In addition, *C. butyricum* intervention significantly alleviated the increase in serum ALB levels in MCB and HCB groups. Histological results showed that hepatic steatosis, lymphocyte aggregation and hepatocyte necrosis induced by CORT were also significantly ameliorated by *C. butyricum* supplementation and the 400 mg/kg *C. butyricum* intervention had the best effects. *C. butyricum* supplementation has been demonstrated to alleviate CCl_4_-induced liver lesions in mice [25]. Meanwhile, other findings have revealed that *C. butyricum* can effectively attenuate the neurohistopathological changes in dementia mice [37], indicating that *C. butyricum* supplementation indeed attenuates CORT-induced liver injury.

Oxidative stress is also an important mechanism of liver injury in animals that can involve increased lipid peroxidation and decreased activities of antioxidant enzymes [38]. Our results revealed that *C. butyricum* attenuated the increase in hepatic MDA and ROS contents and the decrease in SOD and GSH-PX activities induced by CORT in both MCB and HCB groups, indicating that a certain dose of *C. butyricum* may ameliorate liver injury via an anti-oxidative mechanism. MDA has long been considered an indicator of oxidative stress, and the excess production of ROS can induce immunosuppression [39], but *C. butyricum* can enhance the activities of antioxidant enzymes to alleviate oxidative stress in animals. A previous study revealed that *C. butyricum* supplementation can significantly increase the activities of SOD and CAT, and reduce the MDA level after CCl_4_-induced liver injury in mice [25]. Oxidative stress plays an important role in the pathogenesis of CORT [40], and therefore, these key indices can be used to evaluate the status of oxidative stress and thus provide insight into identifying the mechanisms underlying the protective effects of *C. butyricum*.

After a CORT challenge for five days, inflammatory reactions play a crucial role. Inflammatory cytokines, such as IL-1β, IL-6 and TNF-α, are associated with CORT-induced hepatic damage. In agreement with previous reports [23], the pro-inflammatory factors were significantly enhanced after CORT challenge and were alleviated by 400 mg/kg *C. butyricum* supplementation in our study. Moreover, anti-inflammatory factors (IL-4, IL-10 and TGF-β) that were decreased by CORT stimulation were significantly increased in the 400 mg/kg *C. butyricum*-treated groups. The anti-inflammatory mechanism in this liver injury model is complicated, but it at least involves the attenuation of pro-inflammatory expression and an increase in the levels of IL-4, IL-10 and TGF-β.

Non-alcoholic fatty liver disease (NAFLD) is the result of hepatic steatosis because of lipid accumulation in the liver. Extensive efforts have been made to elucidate the mechanisms underlying hepatic steatosis, which include hepatic fatty acid uptake, de novo lipogenesis, β-oxidation and export [41,42]. Fatty liver formation in waterfowl appears very similar to human NAFLD, making Pekin ducks a very interesting model to better understand hepatic steatosis [43,44]. Previous publications reported that *C. butyricum* supplementation alleviated hepatic lipid accumulation by enhancing fatty acid β-oxidation and inhibiting lipogenesis [21,25]. In a recent study, *C. butyricum* attenuated NAFLD progression in broilers, confirming that *C. butyricum* can protect hepatocytes against chronic damage [45]. Of greater interest, the Oil red O stain showed that pre-administration of *C. butyricum* to Pekin ducks receiving CORT significantly inhibited lipid accumulation in the liver and the 400 mg/kg *C. butyricum* intervention showed the best effects. This result indicated that *C. butyricum* can effectively prevent CORT-induced hepatic steatosis in Pekin ducks.

We demonstrated that *C. butyricum* can attenuate CORT-induced oxidative stress, inflammatory responses and hepatic steatosis in Pekin ducks and the 400 mg/kg *C. butyricum* intervention showed the best effects, however, the alleviated mechanisms involved require further exploration. Recently, Illumina transcriptome profiling, as an efficient, rapid method, has become widely used in stock raising [46]. Our study analyzed the hepatic transcriptome of Pekin ducks with 400 mg/kg *C. butyricum* intervention in order to explore the genes and pathways involved in the protective effects of *C. butyricum* against CORT. *C. butyricum* supplementation at 400 mg/kg activated the NF-κB, and toll-like receptor signaling pathways in the liver, thereby promoting lymphocyte development and differentiation, decreased the expression of genes related to pro-inflammatory responses, and increased the expression of genes related to anti-inflammatory responses (such as *Tlr2a*, *Tlr2b* and *Il10*) and anti-oxidant capacity (*Sod3*). Furthermore, the high level of *Alb* expression in both MCB and ANT groups increased the serum ALB levels. These two signaling pathways can regulate inflammatory responses and cytokine secretion [47,48]. PI3K-Akt signaling pathway is involved in inflammation and immune regulation and was also significantly enriched in the liver and the *Il10r-β* and *Il10r-α* genes were enriched in the PI3K-Akt signaling pathway in the MCB group. IL-10 can promote T cell differentiation and proliferation and can induce the secretion of anti-inflammation cytokines, such as IL-4, IL-10, interferon (IFN)-γ, and TGF-β in cytotoxic T cells and NK cells [49,50]. Accordingly, dietary *C. butyricum* supplementation up-regulated the transcription levels of genes involved in cytokine-cytokine receptor interaction (enriched genes: *Lifr*, *Bmpr2*, *Tnfsf10*, *Ccl5*, *Il8*, and *Pdgfra*). Therefore, *C. butyricum* supplementation can improve the antioxidant capacity and inflammatory responses by enhancing the expression of related genes and the enrichment of related pathways to play a hepatoprotective role in Pekin ducks after CORT challenge.

Oil red O staining showed that a CORT challenge induced severe hepatic steatosis, while *C. butyricum* supplementation decreased the accumulation of liver fat and alleviated the degree of hepatic steatosis. At the transcriptional level, we found the expression of *Acsl1*, *Acsl5*, *Cyp7a1*, *Acaa2* and *Pck1* were down-regulated; whereas *Fabp7* and *Cpt1a* were up-regulated in the MCB group. These genes were demonstrated to decrease gluconeogenesis and increase fatty acid β-oxidation, fatty acid transportation and cholesterol metabolism [51,52,53]. These results indicated that *C. butyricum* supplementation in MCB group can modulate hepatic lipid metabolism-related genes in Pekin ducks to alleviate the hepatic steatosis induced by CORT. According to the KEGG analysis, we found that the PPAR signaling pathway, which is associated with lipid metabolism regulation [54], was activated in the *C. butyricum*-treated group in Pekin ducks, indicating that providing *C. butyricum* to Pekin ducks can regulate the expression of lipid metabolism-related genes through activation of the PPAR signaling pathway. In the PPAR signaling pathway, the enriched genes associated with lipid transport promoters (*Apoa1*) and fatty acid oxidation promoters (*Fabp7*, *Cpt1a*, and *Cpt2*) were up-regulated, whereas peroxisome proliferator activated receptor (*Ppar-γ*), gluconeogenesis promoter (*Pck1*), fatty acid transport promoter (*Acsl1/5* and *Lpl*), lipogenesis (*Me1* and *Scd-1*) and cholesterol metabolism (*Cyp7a1*) were down-regulated in the *C. butyricum* supplementation group compared with the CON and ANT groups.

KEGG pathway analysis revealed that *C. butyricum* supplementation activated the glycerophospholipid metabolism signaling pathway and alleviated lipid accumulation, which is consistent with a previous report showing that *C. butyricum* can reduce lipid accumulation in CCl_4_-challenged mice [25]. *Pla2g3* was observed enriched in glycerophospholipid metabolism. *Pla2g3* is the rate-limiting enzyme in glycerophospholipid metabolism [55] and is also involved in signal transduction, immune regulation and the inflammatory response [56,57]. Therefore, *C. butyricum* supplementation modulated glycerophospholipid metabolism by significantly down-regulating *Pla2g3* expression in order to protect the liver from lipid accumulation. Additionally, the adipocytokine signaling pathway also clustered more DEGs (*Socs3*, *Pck1*, *Acsl5* and *Cpt1a*) in the MCB and ANT groups, indicating that compared with antibiotics, feeding *C. butyricum* to ducks may have a more obvious protective effect on alleviating lipid accumulation after CORT challenge. Additionally, *C. butyricum* treatment significantly modulated the expression of genes related to cholesterol transporter activity (up-regulated *Abcg1*, down-regulated *Cetp* and *Stard5*). *Abcg1* can increase the transmembrane transport of sterols and phospholipids [58] and *Cetp* mainly mediates the selective uptake of high density lipoprotein cholesterol by the liver [59]. Therefore, moderate *C. butyricum* intervention increased the transport and decreased the deposition of cholesterol in the liver, which may alleviate hepatic steatosis induced by CORT. In summary, we demonstrated that *C. butyricum* intervention protects Pekin ducks from CORT-induced hepatic steatosis in the liver via the epigenetic modulation of lipogenic and lipophagic genes.

## 5. Conclusions

Our study demonstrated that 400 mg/kg *C. butyricum* intervention can improve growth performance, attenuate body weight loss, and decrease liver function, hepatic oxidative stress, inflammatory reaction, and hepatic lipid accumulation induced by CORT in Pekin ducks. Transcriptome analysis showed that immune system process, metabolism process, and immune response related DEGs were enriched in GO term analysis in the liver to alleviate CORT damage. Moreover, 400 mg/kg *C. butyricum* intervention activated Toll-like receptor signaling pathway (*Tlr2a/b*), PI3K-Akt signaling pathway (*Il10r-α/β*), PPARs signaling pathway (*Cyp7a1*, *Acsl1/5* and *Ppar-γ*), glycerophospholipid metabolism (*Pla2g3*), and cholesterol transporter activity (*Abcg1* and *Cetp*) in the liver, which can improve immune function, promote fatty acid transportation, modulate cholesterol metabolism, and decrease lipid accumulation and fatty acid synthesis following CORT-induced liver injury. The study provides a theoretical basis for the prospective application of *C. butyricum* administration prior to stress exposure in Pekin ducks, provides an optimal dose of *C. butyricum* to select, and indicates that *C. butyricum* may be a kind of protective material or food for animals in order to alleviate stress factors in the breeding process.

## Figures and Tables

**Table 1:** Effect of *C. butyricum* on the liver index of Pekin ducks at 42 d ^1^.

Parameters	CON	LCB	MCB	HCB	ANT	*p* Value
Liver index ^2^ (%)	5.91 ± 0.02	5.84 ± 0.06	5.76 ± 0.07 *	5.74 ± 0.02 *	5.87 ± 0.03	0.003

^1^ Means ± S.D. (N = 12) with superscript * indicates that there are significant differences (*p* < 0.05) compared with the control group. ^2^ liver index = liver weight/body weight.

**Table 2:** The activities of liver function indexes of Pekin ducks at 42 d ^1^.

Parameters ^2^	CON	LCB	MCB	HCB	ANT	*p* Value
AST (U/L)	43.82 ± 28.57	16.63 ± 4.23 *	6.65 ± 2.39 *	25.76 ± 21.05	33.65 ± 16.38	0.012
ALT (U/L)	72.85 ± 42.72	38.08 ± 13.27	13.17 ± 3.98 *	47.25 ± 18.66 *	52.35 ± 9.28	0.002
γ-GGT (U/L)	7.39 ± 6.67	5.84 ± 1.69	2.03 ± 0.80	3.40 ± 1.77	3.57 ± 0.76	0.056
TP (g/L)	32.33 ± 15.04	30.87 ± 10.89	16.29 ± 3.20	31.67 ± 19.52	32.43 ± 5.69	0.142
ALB (g/L)	7.40 ± 1.34	9.73 ± 2.49	7.24 ± 0.91	10.20 ± 1.88 *	10.82 ± 1.90 *	0.004
GLB (g/L)	24.93 ± 15.12	21.14 ± 8.46	9.05 ± 3.69	21.48 ± 18.09	21.62 ± 3.91	0.179

^1^ Means ± S.D. (N = 12) with superscript * indicates that there are significant differences (*p* < 0.05) compared with the control group in the same row. ^2^ AST: glutamic oxalacetic transaminase; ALT: alanine transaminase; γ-GGT: glutamyltransferase; TP: total protein; ALB: albumin; GLB: globulin.

**Table 3:** The oxidative stress levels of Pekin ducks at 42 d of age ^1^.

Parameters ^2^	CON	LCB	MCB	HCB	ANT	*p* Value
T-AOC (U/mg prot)	10.43 ± 1.33	11.72 ± 0.78	12.64 ± 2.87	11.44 ± 2.71	14.41 ± 3.95	0.124
T-SOD (U/mg prot)	92.12 ± 4.07	131.23 ± 10.46 *	161.47 ± 22.51 *	153.97 ± 10.13 *	175.30 ± 13.47 *	<0.001
GSH-PX (U/mg prot)	929.85 ± 73.95	1085.28 ± 189.53	1347.49 ± 97.56 *	1521.46 ± 50.95 *	1377.67 ± 335.80 *	<0.001
MDA (U/mg prot)	8.59 ± 0.67	2.28 ± 0.35 *	2.03 ± 0.44 *	3.12 ± 0.32 *	2.54 ± 0.36 *	<0.001
ROS (F/mL)	672.52 ± 23.69	522.26 ± 22.17 *	485.18 ± 47.44 *	567.18 ± 59.25 *	431.78 ± 16.11 *	<0.001
CORT (ng/mL)	11.75 ± 4.21	8.88 ± 1.17	6.69 ± 2.11 *	7.99 ± 1.14 *	7.25 ± 2.90	0.013

^1^ Means ± S.D. (N = 12) with superscript * indicates that there are significant differences (*p* < 0.05) compared with the control group in the same row. ^2^ T-AOC: total antioxidant capacity; T-SOD: total superoxide dismutase; GSH-PX: glutathione peroxidase; MDA: malonaldehyde; ROS: reactive oxygen species; CORT: corticosterone.

**Table 4:** Effect of *C. butyricum* on inflammation in the liver of Pekin ducks at 42 d ^1^.

Parameters ^2^ (pg/mg prot)	CON	LCB	MCB	HCB	ANT	*p* Value
IL-1β	30.07 ± 13.78	26.24 ± 2.18	26.26 ± 9.42	28.09 ± 9.20	32.41 ± 7.33	0.738
IL-6	17.42 ± 2.30	16.67 ± 4.47	12.99 ± 2.04 *	14.19 ± 1.95	22.25 ± 2.85 *	0.005
TNF-α	70.09 ± 2.52	49.71 ± 6.54 *	69.96 ± 5.69	63.40 ± 16.79	65.61 ± 11.56	0.025
IL-4	29.50 ± 3.58	26.25 ± 3.46	36.05 ± 6.10 *	34.38 ± 6.42	28.12 ± 2.11	<0.001
IL-10	78.82 ± 8.12	85.49 ± 3.83	134.80 ± 16.86 *	123.09 ± 7.24 *	73.02 ± 4.72	<0.001
TGF-β	112.23 ± 12.69	142.57 ± 33.09	152.11 ± 8.13	138.18 ± 31.61	131.95 ± 20.55	0.130

^1^ Means ± S.D. (N = 12) with superscript * indicates that there are significant differences (*p* < 0.05) compared with the control group in the same row. ^2^ TNF-α: tumor necrosis factor-α; TGF-β: transforming growth factor-β.

**Table 5:** RNA-seq data of the nine liver samples used in this experiment ^1^.

Sample	Raw Reads	Clean Reads	Error Rate (%)	Q20 (%)	Q30 (%)	GC Content (%)	Mapped Reads	Mapping Ratio (%)
CON1	69574378	66118288	0.014	97.55	93.10	53.09	44762303	67.70
CON2	86256452	81986216	0.014	97.55	93.10	53.04	57458904	70.08
CON3	63166500	61480000	0.015	97.28	91.61	52.09	44125392	71.77
MCB1	73128306	71283226	0.015	97.39	91.86	51.89	54736639	76.79
MCB2	70861430	69014276	0.015	97.36	91.83	51.46	53077396	76.91
MCB3	68653432	67100512	0.014	97.48	92.08	51.87	50924758	75.89
ANT1	56781984	54956604	0.016	97.16	91.28	51.42	41418070	75.37
ANT2	68298598	66725288	0.015	97.39	91.86	52.78	47897114	71.78
ANT3	83351864	81730498	0.014	97.56	92.30	52.89	56127772	68.67

^1^ GC, guanine-cytosine; Q20, Phred quality score 20; Q30, Phred quality score 30; Mapping ratio, mapped reads/all clean reads.

**Table 6:** Enriched KEGG pathways analysis in MCB vs CON and MCB vs ANT.

KEGG Pathway	Gene Counts	*p* Value
MCB vs CON		
Cell adhesion molecules (CAMs)	8	0.0025
Cytokine-cytokine receptor interaction	5	0.0242
PI3K-Akt signaling pathway	5	0.0012
Metabolism of xenobiotics by cytochrome P450	4	0.0031
PPAR signaling pathway	4	0.0219
Glutathione metabolism	3	0.0340
Toll-like receptor signaling pathway	3	0.0238
MCB vs ANT		
Glycerophospholipid metabolism	8	0.0003
AMPK signaling pathway	6	0.0115
Cytokine-cytokine receptor interaction	6	0.0121
PI3K-Akt signaling pathway	6	0.0540
PPAR signaling pathway	5	0.0042
Adipocytokine signaling pathway	4	0.0135
Glycerolipid metabolism	4	0.0320
NF-kappa B signaling pathway	3	0.0202
Fatty acid biosynthesis	2	0.0127

**Table 7:** Significantly enriched pathways for differential expressed genes.

Pathway Description	Enriched Genes	*P* Value
MCB vs CON		
Cell adhesion molecules (CAMs)	*Itga6, Tead4, Mpzl1, Cd2, Ptprf, Cd226, Alcam, Loc480788*	0.0025
PI3K-Akt signaling pathway	*Rpl35, Ccnd2, Il10r-β, Il10r-α, Csf2ra*	0.0096
PPAR signaling pathway	*Apoa1, Fabp1, Cyp7a1, Lpl, Me1*	0.0219
Complement and coagulation cascades	*Mbl2, C6, C9, Cr1*	0.0310
MCB vs ANT		
Glycerophospholipid metabolism	*Gpat, Lpin1, Gnpat, Pla3g3, Lpgat1, Agpat3, Lcat, Agpat2*	0.0003
AMPK signaling pathway	*Fas, Pck1, Pparg, Cpt1a, Ccna2, Hmgcr*	0.0115
Cytokine-cytokine receptor interaction	*Lifr, Bmpr2, Tnfsf10, Ccl5, Il8, Pdgfra*	0.0121
PPAR signaling pathway	*Fabp7, Acsl5, Pck1, Pparg, Cpt1a*	0.0042
Adipocytokine signaling pathway	*Socs3, Pck1, Acsl5, Cpt1a*	0.0135

## Supplementary Materials

The following are available online at https://www.mdpi.com/2076-2607/7/9/358/s1, Table S1: Ingredients and nutrient levels of the basal diets (air-dry basis), Table S2: Primers information used for qRT-PCR in this study, Table S3: The top-15 genes expressed in both MCB and ANT groups, respectively, Table S4: The data of CORT levels in serum of Pekin ducks at 37 d before CORT, Figure S1: The numbers of exons and introns, Figure S2: The GO annotation of the gross unigenes in the hepatic transcriptome, Figure S3: The KEGG classification of the gross unigenes in the hepatic transcriptome, Supplementary file 1: Detailed information of DEGs, Supplementary file 2: Detailed information of GO enrichment of DEGs, Supplementary file 3: Detailed information of KEGG enrichment of DEGs, Supplementary file 4: The raw data of qRT-PCR, Supplementary file 5: PAS value annotation.
