# Supplementary material for: A Discovery of Relevant Hepatoprotective Effects and Underlying Mechanisms of Dietary Clostridium butyricum Against Corticosterone-Induced Liver Injury in Pekin Ducks
Source: Microorganisms. 2019 Sep 16;7(9):358. doi: 10.3390/microorganisms7090358 (PMC6780423; doi:10.3390/microorganisms7090358)
Supplement: Supplementary file 1 [file microorganisms-07-00358-s001.zip › Figure S1-S3.docx]

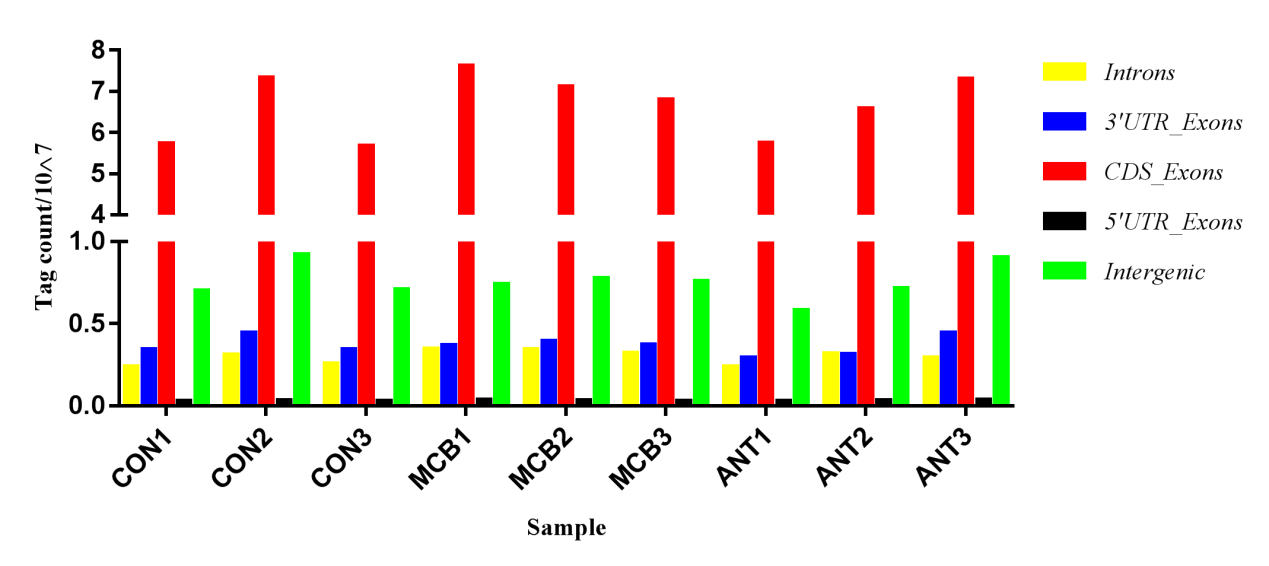


**Figure S1** The numbers of exons and introns. Tag count is the number of reads mapped to the regions of exons and introns in the Anas platyrhynchos genome. CDS, coding DNA sequence.


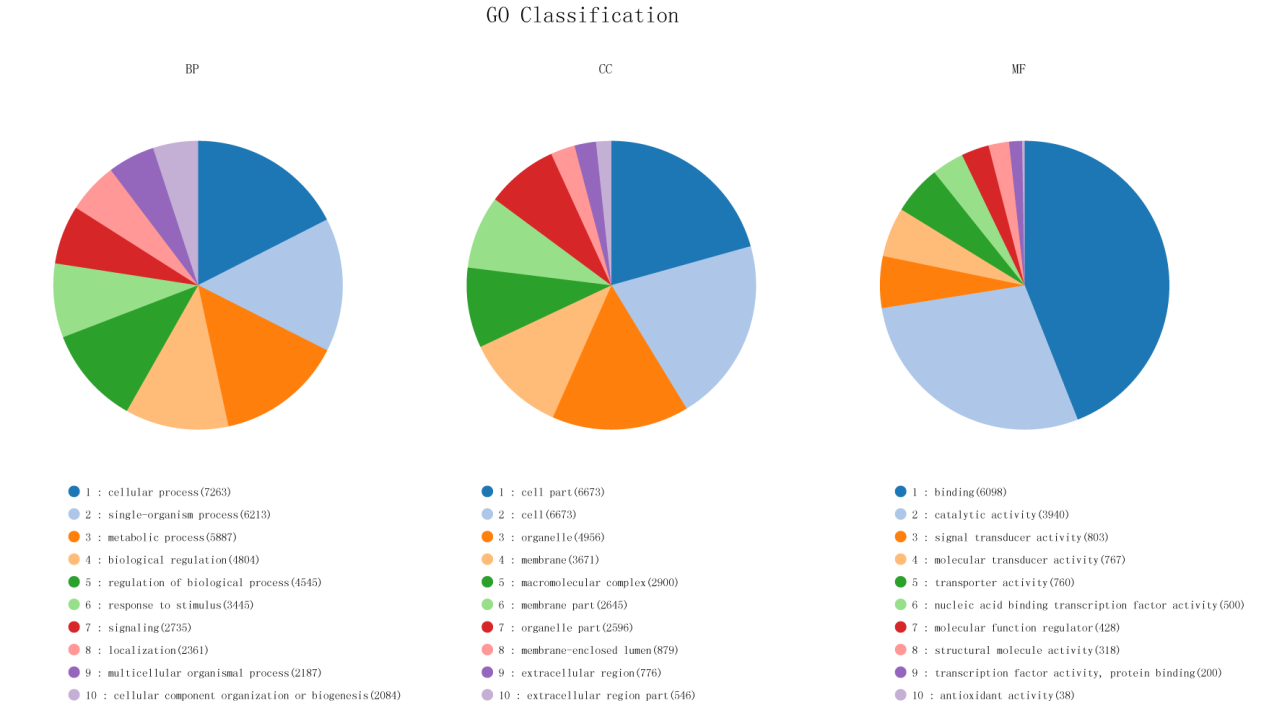


**Figure S2** The GO annotation of the gross unigenes in the hepatic transcriptome.


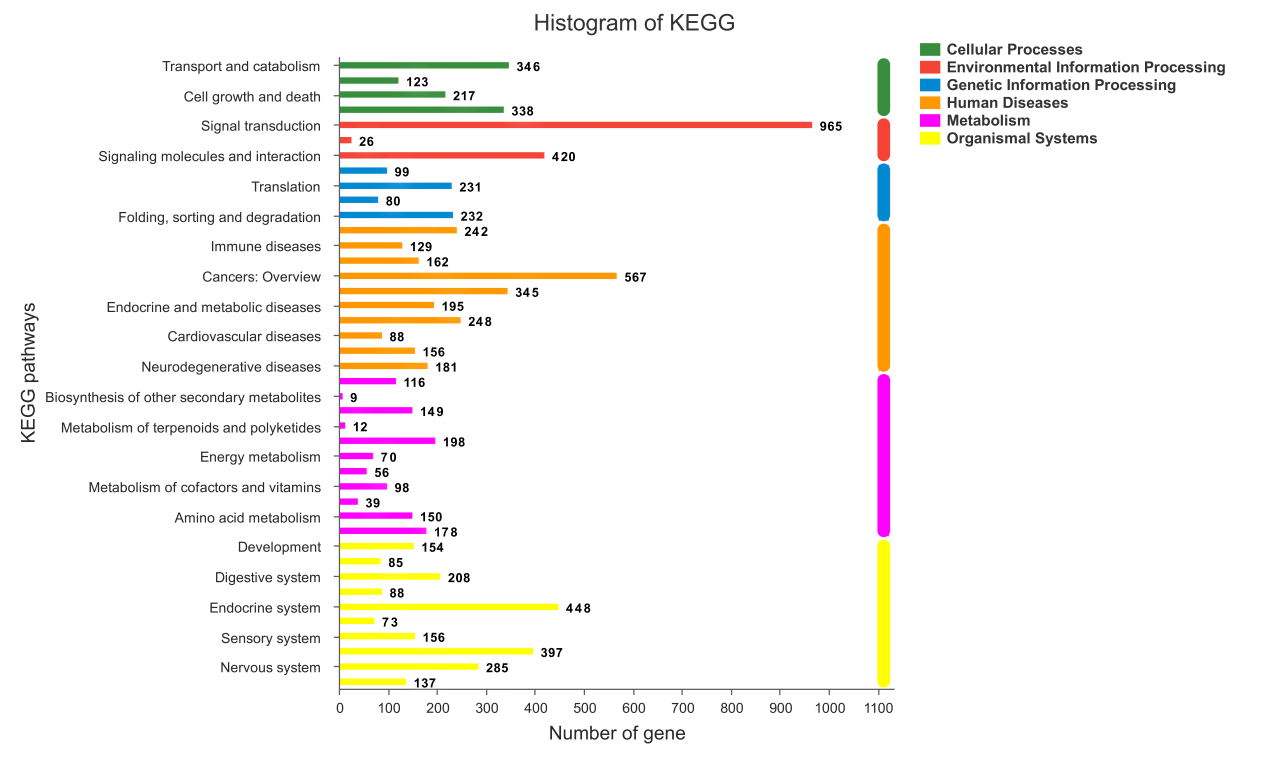


**Figure S3** The KEGG classification of the gross unigenes in the hepatic transcriptome.
