# Supplementary material for: A Discovery of Relevant Hepatoprotective Effects and Underlying Mechanisms of Dietary Clostridium butyricum Against Corticosterone-Induced Liver Injury in Pekin Ducks
Source: Microorganisms. 2019 Sep 16;7(9):358. doi: 10.3390/microorganisms7090358 (PMC6780423; doi:10.3390/microorganisms7090358)
Supplement: Supplementary file 1 [file microorganisms-07-00358-s001.zip › Supplementary file 5 PAS value annoation.docx]

**What is** **PAS Z score?**

PAS value: The Pathway Activation Strength value, served as the activation profiles of the signaling pathways based on the expression of individual genes.


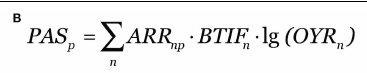


Here the case-to-normal ratio, CNRn, is the ratio of expression levels for a gene n in the sample under investigation to the same average value for the control group of samples. The Boolean flag of BTIF (beyond tolerance interval flag) equals zero when the CNR value has passed simultaneously the two criteria that demark the significantly perturbed expression level from essentially normal. The first criterion is the expression level for the sample lies within the tolerance interval, where p > 0.05. The second criterion is the discrete value of ARR (activator/repressor role) equals to the following fixed values: -1, when the gene/protein n is a repressor of pathway excitation; 1, if the gene/protein n is an activator of pathway excitation; 0, when the gene/protein n can be both an activator and a repressor of the pathway; 0.5 and -0.5, respectively, if the gene/protein n is rather an activator or repressor of the signaling pathway p, respectively.

Z-score is the coordinate value of the standard normal distribution. The coordinate value of the standard normal distribution is a probability distribution that is important in mathematics, physics, engineering, etc., and has significant influence in many aspects of statistics.

Differentially enriched GO processes were identified according to their reporter score from the Z-score of individual genes. A reporter score of Z = 1.96 or Z = -1.96 was used as a detection threshold for significantly different processes.
