# Supplementary material for: A Discovery of Relevant Hepatoprotective Effects and Underlying Mechanisms of Dietary Clostridium butyricum Against Corticosterone-Induced Liver Injury in Pekin Ducks
Source: Microorganisms. 2019 Sep 16;7(9):358. doi: 10.3390/microorganisms7090358 (PMC6780423; doi:10.3390/microorganisms7090358)
Supplement: Supplementary file 1 [file microorganisms-07-00358-s001.zip › Table S1-S4.docx]

| **Table S1** Ingredients and nutrient levels of the basal diets (air-dry basis) | | |
| --- | --- | --- |
| Items | 1-21d | 22-42d |
| Ingredients (%) |  |  |
| Corn | 56.00 | 60.24 |
| Soybean meal | 32.69 | 24.67 |
| Wheat middling | 5.00 | 9.00 |
| Soybean oil | 2.10 | 1.80 |
| Phytase | 0.02 | 0.02 |
| Dicalcium phosphate | 1.00 | 1.60 |
| Limestone | 1.50 | 1.20 |
| DL-Methionine | 0.15 | 0.12 |
| L-Lysine | 0.20 | 0.10 |
| Vitamin premix^1^ | 0.02 | 0.02 |
| Trace mineral premix^2^ | 0.20 | 0.20 |
| NaCl | 0.35 | 0.30 |
| Choline chloride (50%) | 0.24 | 0.20 |
| Santoquin (33%) | 0.03 | 0.03 |
| Maifanite | 0.50 | 0.50 |
| Total | 100 | 100 |
| Nutrient levels^3^ (%) |  |  |
| AME (MJ/kg) | 12.31 | 12.53 |
| Crude protein (%) | 19.52 | 16.83 |
| Lysine (%) | 1.12 | 0.87 |
| Methionine (%) | 0.46 | 0.39 |
| Calcium (%) | 0.88 | 0.89 |
| Available phosphorus (%) | 0.39 | 0.29 |
| Total phosphorus (%) | 0.54 | 0.62 |
| Methionine+Cysteine (%) | 0.79 | 0.69 |
| ^1^The vitamin premix provided the following per kilogram of diet: vitamin A, 12,500 IU; vitamin D_3_, 3500 IU; vitamin E, 20 IU; vitamin K_3_, 2.65 mg; thiamin, 2.00 mg; riboflavin, 6.00 mg; pyridoxin, 3.00 mg; VB_12_, 0.025 mg; biotin, 0.0325 mg; folic acid, 12.00 mg; pantothenic acid, 50 mg; nicotinic acid, 50.00 mg.  ^2^The mineral premix provided the following per kg of diet: Cu, 6 mg; Fe, 80 mg; Zn, 40 mg; Mn, 100 mg; Se, 0.15mg; I, 0.35 mg.  ^3^ Calculated values. | | |

| **Table S2** Primers information used for qRT-PCR in this study | | |
| --- | --- | --- |
| Genes | Forward primer sequence (5’→3’) | Reverse primer sequence (5’→3’) |
| *TLR2A* | TCTGAGGAACAAGCCCTGAAGC | CAGGACGAGGATGACCAGGAAA |
| *IL10* | CGGCACAGAAATCCCAGAGC | CAGCAGGCTCACAAAATAAACATCT |
| *SOD3* | GGATGACTTACGCCGCTTGTGA | CTCGTGGATGTGGATCGCTCTG |
| *TLR2B* | ATGAGCGACAGCGGCGAG | TTAGTAGCGACGAGGTGA |
| *FABP* | CCACACTGCGTTGTACCTTCCA | CGTCACCACAAAGTCGTCTCCT |
| *ATP6V0D2* | ATGGGGCCTTGCAAAAGAAA | GCTAACAACCGCAACCCCTC |
| *ACSL1* | ATCTGGTGGAACGAGGCAAG | TCCTTTGGGGTTGCCTGTAG |
| *CYP7A1* | CTGGGCTTCACAGGCTAACACC | TTCAGTGTGGGGTCGTTGGG |
| *TRIM35* | GATCTTTTTGAGAAATGGAAGCAGG | TATTGAACTGAATTTATTGTCACGTGTC |
| *IL22RA2* | TGGATTTGCTGTTTATGTCTCT | CTTCCCATCTTCCTTTTGGTTA |
| *CPT1A* | ACGCCGTGAAGTATAACCCT | CCAAAAATCGCTTGTCCCTT |
| *FABP7* | TGATGAGACCACAGCAGATGACAG | GTTCCCATCCACCACTTTCCTCTT |
| *ILDR1* | GTGGTGGGATCATGGCGTAT | TTGCATGTTTTCAGCCCATCA |
| *IGFBP4* | GGGTGTTCTCTTTGGTGTTA | TGTTTTTAGGTGGGTGGATG |
| *ACSL5* | TTTTTGTACACGGGGAGAGC | ACAGGCTGTCAATTTGGGTC |
| *PPARG* | CCTTCCCCACCCTATTTTTC | GCTTCTCCTTCTCCGCTTGT |
| *FASN* | ATAGTGGAGGCTCTGGCAGGAA | GCAATCGGTGGCAATGTAGTCC |
| *IL17B* | GGTCCTCCTGCATGCTGAAG | ACTGGCCTCACAACCTGCTG |
| *HSPB3* | CAATGTTGCCAAGCCTGATCTGA | GCATCCAGTTCAATCCACGAGTT |
| *GAPDH* | GGTAGTGAAGGCTGCTGCTGATG | CCACCACACGGTTGCTGTATCC |

| Table S3 The top-15 genes expressed in both MCB and ANT groups, respectively | | | | |
| --- | --- | --- | --- | --- |
| Gene_name | Full_name | *P*-value | MCB_FPKM | ANT_FPKM |
| Alb | Albumin | 0.0216 | 73622.26 | 73650.22 |
| Ttr | Transthyretin | 0.0082 | 17239.27 | 12792.74 |
| Thrsp | Mid1-interacting protein 1-B-like | 0.0011 | 16864.95 | 6117.85 |
| Hpx | Hemopexin | 0.6783 | 16064.11 | 19032.18 |
| Apoa1 | Apolipoprtein A1 | 0.0280 | 15505.54 | 24974.57 |
| Ahsg | Alpha 2-HS glycoprotein | 0.7265 | 13987.47 | 15905.36 |
| Mt1 | Metallothionein 1 | 3.56E-06 | 8949.72 | 26342.59 |
| Eef1a1 | Eukaryotic translation elongation factor 1 alpha 1 | 0.1445 | 6754.11 | 8607.16 |
| Vtn | Vitronectin | 0.7265 | 5996.72 | 6089.99 |
| Fabp1 | Fatty acid-binding protein, liver | 0.6538 | 5854.19 | 6505.66 |
| Ppdpf | Pancreatic progenitor cell differentiation and proliferation factor | 0.2053 | 5693.30 | 7028.09 |
| Fgg | Fibrinogen gamma chain | 0.0514 | 5588.37 | 8824.39 |
| Aldob | Aldolase, fructose-bisphosphate B | 0.0022 | 4999.47 | 10215.94 |
| Fgb | Fibrinogen beta chain | 0.0425 | 4917.75 | 8018.83 |
| Scd | Stearoyl-CoA desaturase | 0.0002 | 4686.23 | 2392.88 |
| Note: FPKM, fragments per kilobase of exon per million fragments mapped. | | | | |

| **Table S4** The data of CORT levels in serum of Pekin ducks at 37 d before CORT^1^ | | | | | | |
| --- | --- | --- | --- | --- | --- | --- |
| Parameters | CON | LCB | MCB | HCB | ANT | *p* value |
| CORT^2^ (ng/ml) | 6.47±0.62 | 6.47±0.30 | 6.58±0.52 | 6.35±0.50 | 6.76±0.61 | 0.714 |
| ^1^Means ± S.D. (N=6) with superscript * indicates that there are significant differences (*p* < 0.05) compared with the control group.  ^2^CORT: corticosterone. | | | | | | |
